# Gray Wolves as Climate Change Buffers in Yellowstone

**DOI:** 10.1371/journal.pbio.0030092

**Published:** 2005-03-15

**Authors:** Christopher C Wilmers, Wayne M Getz

**Affiliations:** **1**Department of Environmental Science, Policy and ManagementUniversity of California, Berkeley, CaliforniaUnited States of America; **2**Mammal Research Institute, Department of Zoology and EntomologyUniversity of PretoriaSouth Africa; Princeton UniversityUnited States of America

## Abstract

Understanding the mechanisms by which climate and predation patterns by top predators co-vary to affect community structure accrues added importance as humans exert growing influence over both climate and regional predator assemblages. In Yellowstone National Park, winter conditions and reintroduced gray wolves *(Canis lupus)* together determine the availability of winter carrion on which numerous scavenger species depend for survival and reproduction. As climate changes in Yellowstone, therefore, scavenger species may experience a dramatic reshuffling of food resources. As such, we analyzed 55 y of weather data from Yellowstone in order to determine trends in winter conditions. We found that winters are getting shorter, as measured by the number of days with snow on the ground, due to decreased snowfall and increased number of days with temperatures above freezing. To investigate synergistic effects of human and climatic alterations of species interactions, we used an empirically derived model to show that in the absence of wolves, early snow thaw leads to a substantial reduction in late-winter carrion, causing potential food bottlenecks for scavengers. In addition, by narrowing the window of time over which carrion is available and thereby creating a resource pulse, climate change likely favors scavengers that can quickly track food sources over great distances. Wolves, however, largely mitigate late-winter reduction in carrion due to earlier snow thaws. By buffering the effects of climate change on carrion availability, wolves allow scavengers to adapt to a changing environment over a longer time scale more commensurate with natural processes. This study illustrates the importance of restoring and maintaining intact food chains in the face of large-scale environmental perturbations such as climate change.

## Abstract 2

Reintroducing wolves can help ameliorate the negative effects of warmer winters on other species and reveals the importance of maintaining intact food chains in the face of climate change

## Introduction

Average earth temperatures have increased by 0.6 °C over the last 100 years [1] and are predicted to increase by 1.4–5.8 °C over the next century [2]. Commensurate with rising global temperatures are regional changes in weather patterns affecting the quantity and timing of precipitation and moisture levels. A challenge facing ecologists is to understand how these changes in the abiotic environment will impact populations and communities of organisms. Already, studies have documented the effect of a changing climate on the phenology, range, reproductive success, and synchrony of certain plants and animals (see [1] for a comprehensive review). In addition, climate-caused community-level changes have been documented when range shifts lead to the transfer of an entire assemblage of species [3].

Given such responses by individual species, we can expect consequent shifts in trophic structure and competitive hierarchies at the community scale [4]. Studies addressing this problem have focused primarily on how species-specific responses in phenology and geographic range alter competitive balances and the timing of food availability for neonates [5,6,7,8]. In Britain, for instance, winter warming has precipitated disparate responses in the breeding phenology of different amphibian species, exposing frog larvae *(Rana temporaria),* which have shown no phenological response, to higher levels of predation from newts (*Triturus* spp.) that are entering ponds earlier than before [5].

As predicted by community stability theory, the impact of climate change on communities may vary in relation to levels of species diversity [9,10,11,12]. Depauperate communities or those lacking keystone species [13,14] may be more vulnerable to the perturbing effects of climate change than more speciose communities. As such, understanding the mechanisms or pathways that confer community resistance to climate change will be important to conservationists and managers in mitigating the effects of a changing climate on shifting community patterns and local extinctions.

The reintroduction of gray wolves *(Canis lupus)* to Yellowstone National Park (NP) in 1995 [15] provides a research opportunity for comparing the response of an ecosystem to climate change in scenarios with and without direct human alteration of species composition. Wolf restoration is already realizing a change on the Yellowstone ecosystem by altering the quantity and timing of carrion availability to scavengers [16]. Ravens *(Corvus corax),* bald eagles *(Haliaeetus leucocephalus),* golden eagles *(Aquila chrysaetos),* magpies *(Pica pica),* coyotes *(Canis latrans),* grizzly bears *(Ursus arctos),* and black bears *(Ursus americanus)* are each frequent visitors at wolf kills [17] and are highly reliant on winter carrion for survival and reproductive success [16,18,19,20,21,22].

Prior to wolf reintroduction, winter mortality of elk *(Cervus elaphus),* the most abundant ungulate in Yellowstone, was largely dependent on snow depth (SDTH) [23]. Deep snows lead to increased metabolic activity [24] and decreased access to food resources, thereby causing elk to weaken and die [25]. In the absence of wolves, carrion was plentiful both during severe winters and at the end of moderate winters, but more scarce in early winter or during mild winters [23]. Reintroduced wolves are now the primary cause of elk mortality throughout the year [26]. Scavengers that once relied on winter-killed elk for food now depend on kleptoparasitizing wolf-killed elk [16]. Hence carrion availability has become primarily a function of wolf pack size, with SDTH an important but secondary factor.

As global temperatures rise, evidence suggests that northern latitude and high elevation areas will experience shorter winters and earlier snow melts [27]. Given the overwhelming influence of gray wolves on scavenger food webs, community-level responses to climatic changes in the absence of wolves may differ substantially from those in the presence of Yellowstone's newly restored top carnivore. As such, we analyzed over 50 y of weather data from Yellowstone's northern range for trends in winter conditions, and constructed empirically and dynamically grounded scenarios to investigate how changes in SDTH and seasonality differentially affect scavengers in the presence and absence of wolves.

## Results

### Weather Data Analysis

Over the past 55 y, average monthly SDTH at the Mammoth Hot Springs weather site show a steady decline in all winter months except November [the effect is significant at *p* ≤ 0.05 for February through April and nearly significant for December and January (Figure 1)]. Furthermore, the slope of the line relating SDTH to year becomes more negative with each month, indicating a more pronounced effect of climate change in late winter. The result for April, however, is confounded by a number of zeros, which created a violation of the normality assumption for the linear regression. Average monthly SDTH at the Tower Falls weather site (Figure 2) did not indicate a strong pattern in the early winter, but showed a significant decline in the late-winter months of March and April (Figure 2E and 2F).

**Figure 1:**
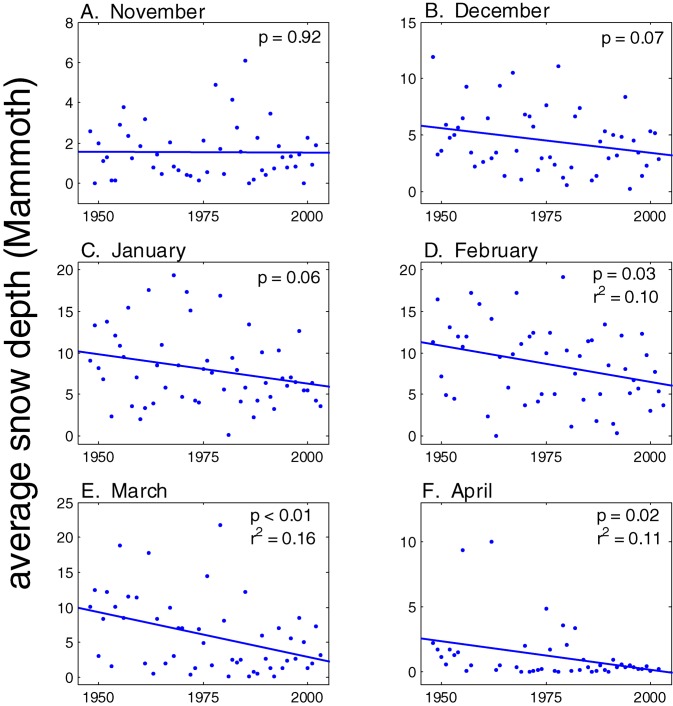
Winter Snow Depths 1948–2003 at Mammoth Hot Springs Average monthly SDTH for November (A), December (B), January (C), February (D), March (E), and April (F) 1948–2003 at the Mammoth Hot Springs weather site.

**Figure 2:**
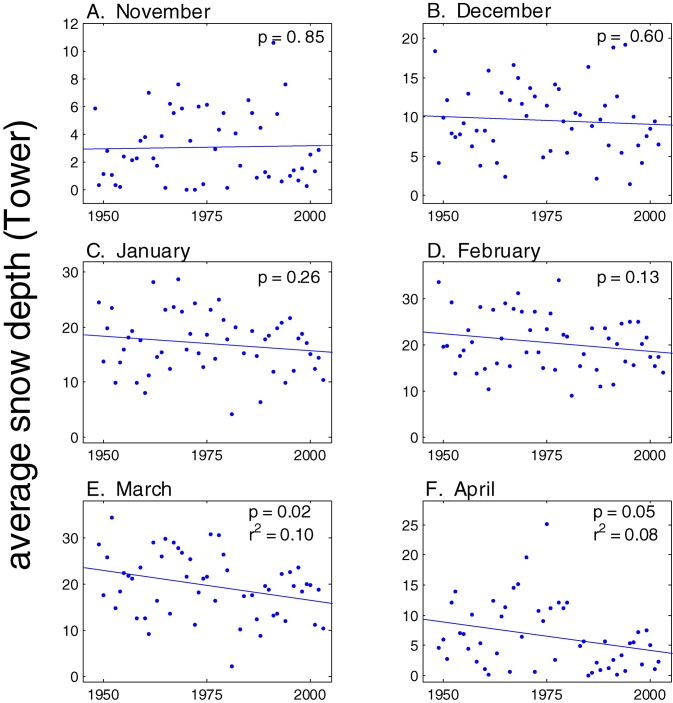
Winter Snow Depths 1948–2003 at Tower Falls Average monthly SDTH for November (A), December (B), January (C), February (D), March (E), and April (F) 1948–2003 at the Tower Falls weather site.

Winters in Yellowstone are getting shorter. While we did not detect a difference in the date of the arrival of the first snow, we did detect a declining trend in the date of last snow on the ground (Figure 3A and 3B).

**Figure 3:**
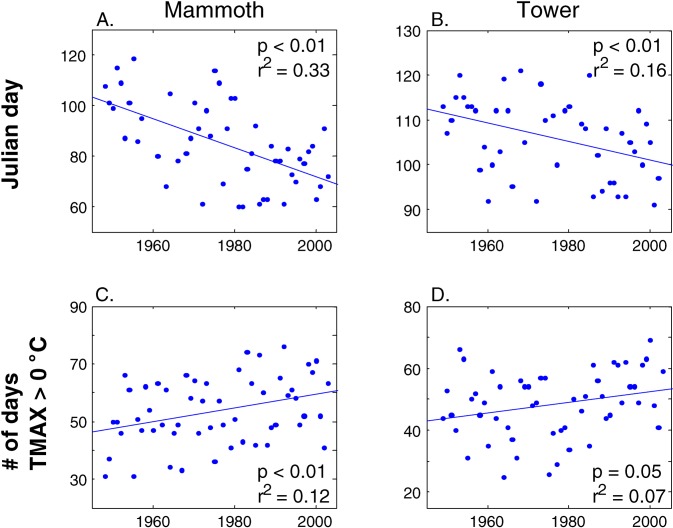
Changes in the Last Day of Snow Cover over the Last 55 Years at Mammoth Hot Springs and Tower Falls Last day of snow cover is reported as the number of days from January 1 of that year until the first day of bare ground. Changes in last day of snow cover over the last 55 y are shown for Mammoth Hot Springs (A) and Tower falls (B). The number of days from January through March that temperatures exceeded freezing at Mammoth (C) and Tower (D) are increasing with time.

At both the Tower and Mammoth weather sites, the number of days that maximum temperature (TMAX) exceeded freezing for the period of January through March increased significantly (Figure 3C and 3D). Furthermore, midwinter snowfall is decreasing, and late-winter minimum temperature (TMIN) and TMAX show signs of increasing in certain months (Table 1).

**Table 1:**
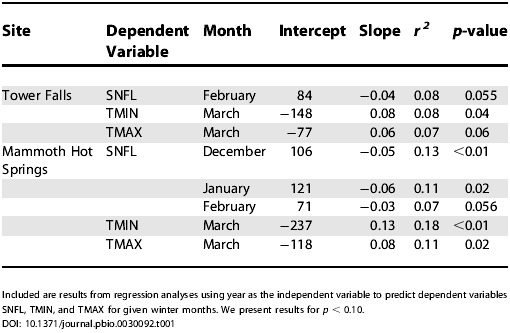
Regression Analyses Predicting Mean Monthly SNFL, and Average Late-Winter TMIN and TMAX

Included are results from regression analyses using year as the independent variable to predict dependent variables SNFL, TMIN, and TMAX for given winter months. We present results for *p* < 0.10

### Wolf Effects

#### Statistical model.

The presence of wolves in Yellowstone significantly mitigates the reduction in late-winter carrion expected under climate change (Figure 4). In the scenario without wolves, late-winter carrion availability is reduced by 27% in March and by 66% in April. In contrast, the scenario with wolves reveals a reduction in carrion availability of only 4% in March and 11% in April. There was not a significant difference in the reduction of early- to midwinter carrion (December through February) between the two scenarios.

**Figure 4:**
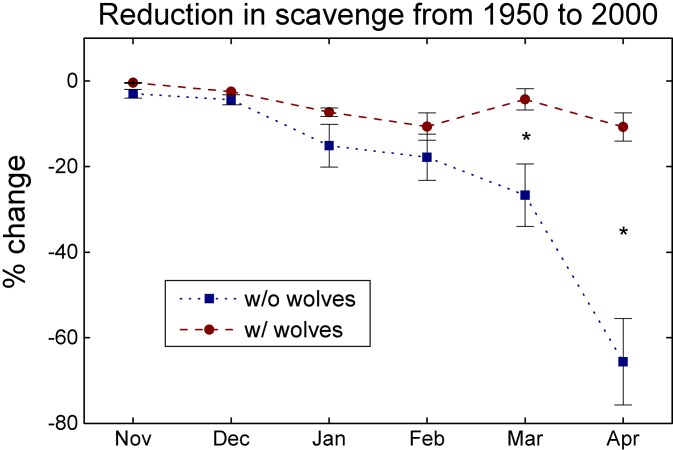
Reduction in Winter Carrion Available to Scavengers due to Climate Change 1950–2000: Statistical Model Shown are percent reductions (± standard error) in winter carrion available to scavengers due to climate change from 1950 to 2000 with and without wolves in our statistical model. * Significant difference between the two scenarios.

#### Dynamic model.

Percent change, *z,* in late-winter carrion from 1950 to 2000 was not sensitive to changes in any of the parameters in either scenario with or without wolves. Specifically, *r* ^2^ values did not exceed 0.02 for any of the parameters regressed upon *z.* Mean monthly percent change in carrion availability from 1950 to 2000 under scenarios with and without wolves reveals a relative reduction in late-winter carrion from 1950 to 2000 and an increase in early-winter carrion (Figure 5). Note that this change in carrion availability is much less pronounced in the presence than in the absence of wolves.

**Figure 5:**
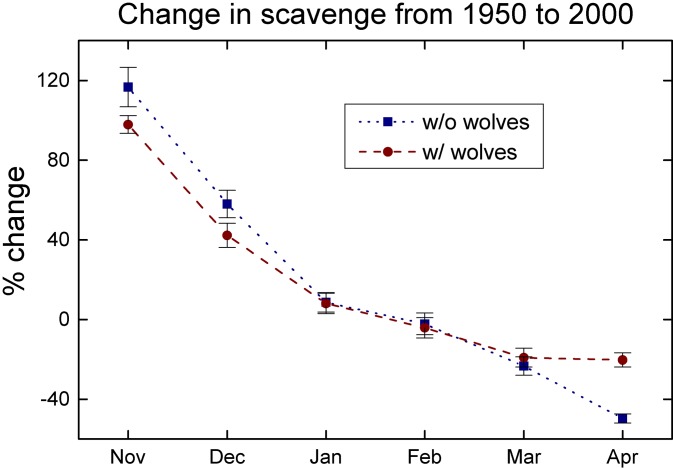
Change in Carrion Available to Scavengers due to Climate Change 1950–2000: Dynamic Model Shown is the mean monthly change (± standard error) in carrion available to scavengers due to climate change from 1950 to 2000 with and without wolves in our dynamic model.

## Discussion

The winter period on the northern range of Yellowstone NP is shortening. Both late-winter SDTHs and the overall duration of snow cover have decreased significantly since 1948 (see Figures 1–3). There are several potential causes of reduced snow pack. Average TMIN and TMAX values are increasing in late winter, while midwinter snowfall appears to be declining (Table 1). Compounding the effects of declining snowfalls on SDTH is an increase in the number of winter days with temperatures above freezing (see Figure 3C and 3D).

Decreases in late-winter snow pack and in the date of last snow cover imply that elk will recover sooner from the detrimental stresses of winter: Smaller snow packs allow elk easier access to food and decrease energy expenditures required for movement. In addition, herbaceous plant growth usually begins within a few days to weeks of last snow cover [28], so elk may increase the quality and quantity of food intake earlier in the year, thus shortening the physiologically stressful winter period. These factors are likely to influence the timing and abundance of carrion as late-winter elk mortality declines. As we demonstrate here, climate change serves to sharply reduce the amount of late-winter carrion available to Yellowstone's scavengers (see Figure 4). According to our statistical and dynamic models, however, this reduction is much less pronounced in the presence of wolves. In our statistical model, for instance, we found an 11% reduction with wolves versus a 66% reduction without wolves in April (see Figure 4). Our dynamic model, which incorporates wolf and elk population growth, also reveals a decline in late-winter carrion, especially in the absence of wolves (Figure 5). In contrast to the statistical model, our dynamic model predicts an increase in early winter carrion, but less so with wolves. As the winter period shortens, elk that normally would die in March and April will increasingly die in the early winter months, November through February. This will lead to an increasingly pulsed or seasonal carrion resource. It is important to note that our model has more detailed elk than wolf dynamics. As suitable data become available, future work can attempt to tease out such factors as the effects of SDTH and territoriality on wolf kill-rate. In both our dynamic and statistical models we find that wolves buffer the effects of climate change on carrion abundance and timing.

This effect will be crucial to scavenger species in the Yellowstone area that are highly dependent on winter and spring carrion for overwinter survival and reproduction. Under scenarios without wolves, these species could face food bottlenecks in the absence of late-winter carrion. The magnitude of this effect will depend on how quickly these species adapt to a changing environment and how their other food resources respond to a shortening of the winter period.

Asynchrony of organismal responses to climate change has been prevalent in other areas, leading to changes in the competitive balance between species and to food shortages at important times of year [1]. Yellowstone should prove no exception. Species that respond to weather cues, such as many herbaceous plants, will simply start growing earlier in the year in response to earlier snow melt. Species that respond primarily to day length cues, such as some hibernating species, may change less. Coyotes, for instance, are highly dependent on late-winter and early-spring carrion to carry them over until late spring, when elk calves and ground squirrels become abundant. If late-winter carrion were to disappear without a corresponding change in the timing of elk calving or ground squirrel emergence, a serious food bottleneck could develop.

As carrion becomes more concentrated over a shorter window of the year, the relative access to carrion among different scavenger species may change. Highly aggregated or pulsed resources saturate local communities of scavengers, allowing species with better recruitment abilities (animals capable of covering large distances and communicating about the location of resources such as ravens and bald eagles) to dominate consumption at carcasses [17]. Resources that are more dispersed, conversely, do not saturate local scavenger communities, so that a competitive dominance hierarchy (with grizzly bears and coyotes at the top) determines which species consume the bulk of available scavenge. Our analysis suggests that winter carrion in the absence of wolves will become increasingly pulsed during winter. Consequently, areas without wolves may experience an increase in scavengers with high recruitment abilities. Actual numerical responses by scavenger species to wolf-provided carrion can now be tested in field studies by comparing areas with wolves to those without wolves in order to determine if changes in scavenger population sizes following wolf reintroduction are consistent with the predicted magnitude of the temporal subsidy due to wolves.

As the climate warms, those species will persist that are able to adapt to differences in the environment. Late-winter carrion in Yellowstone will decline with or without wolves, but by buffering this reduction, wolves extend the timescale over which scavenger species can adapt to the changing environment. It is important to note that under present-day climatic conditions, we expect wolves to decrease the long-term average elk population in Yellowstone [29]. This will lead to a corresponding decrease in average yearly carrion levels, which is expected to be small, however, because declines in carrion due to a drop in elk numbers will be partly offset by a higher turnover in the elk population due to wolf predation on old animals [29]. Scenarios both with and without wolves therefore provide a meaningful and roughly equivalent (see Figure 4 in [29]) amount of carrion to scavengers. What we demonstrate here is that scavengers in areas without wolves will experience carrion as an increasingly pulsed resource under climate change, whereas in areas with wolves carrion will remain spread out over the winter months.

The primary objective of this study is to understand the influence of winter climate and predation on trophic dynamics. Our analysis is retrospective, examining what would have happened to scavenge availability in scenarios with and without wolves over the last fifty years of climate change. One may ask, however, what these results imply in light of predictions for continuing global warming into the future. Elk population numbers in Yellowstone are currently constrained by the availability of winter range, where snow levels are low enough to allow for elk movement and cratering through the snow to access food resources. If snow levels in Yellowstone continue to decline in the future, winter range expansion and thus higher elk densities are likely to occur. We expect, therefore, that the wolf-elk-scavenger complex will accrue added importance in the years to come. Future studies examining climate change impacts on spring and summer rainfall, which sets forage levels for elk, will be crucial to further deciphering the effects of global change on trophic relationships in Yellowstone.

We are just beginning to understand the interaction between top predators, such as wolves, and global climate patterns. On Isle Royale, trophic effects have recently been shown to be mediated by behavioral responses to climate. There, gray wolf pack size is partly controlled by climatic conditions that, in turn, affect wolf kill-rates on moose *(Alces alces)* and consequent herbivory levels on balsam fir *(Abies balsamea)* [30]. In Yellowstone, our scenarios demonstrate that wolves act to retard the effects of a changing climate on scavenger species. Together these results begin to elucidate the expected changes that may occur to boreal ecosystems as a result of climate change effects on top predators.

## Materials and Methods

### 

The northern range of Yellowstone NP is the wintering area of the park's largest elk herd and home to 4–6 gray wolf packs. Elevations range from 1,500 to 3,400 m, with 87% of the area between 1,500 and 2,400 m [25]. The climate is characterized by short, cool summers and long, cold winters, with most annual precipitation falling as snow. Mean annual temperature is 1.8 °C, and mean annual precipitation is 31.7 cm [25]. Large, open valleys of grass meadows and shrub steppe dominate the landscape, with coniferous forests occurring at higher elevations and on north-facing slopes.

#### Weather data analysis

Since 1948, meteorological data has been collected daily from two permanent weather stations on the northern range of Yellowstone NP. One is located in Mammoth Hot Springs at park headquarters near the northern entrance to the park. The other is located at the Tower Falls ranger station about 29 km east of Mammoth. Data for the period 01 August 1948 to 01 June 2003 were made available to us by the Western Regional Climate Center in Reno, Nevada, United States.

Using linear regression, we investigated multiannual trends in monthly average SDTH over the 55 y provided in the data set. SDTH is treated as the response variable and regressed upon year. We also examined trends in the timing of the date of first bare ground. This was defined as the first day of the year for which SDTH was zero. In order to understand changing patterns in SDTH, we analyzed average monthly snowfall (SNFL), average TMIN and TMAX, and the number of days per winter that TMAX exceeded freezing.

#### Wolf effects: Statistical model

In order to compare the effects of carrion availability to scavengers under climate change in scenarios with and without wolves, we used previously published regression equations [23] relating SDTH, *S,* to monthly carrion availability, *C_p_,* prior to wolf reintroduction given by



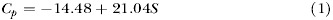



and relating SDTH and wolf pack size to carrion availability, *C_a_,* after wolf reintroduction [16] obtained using



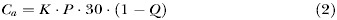



where *K* is the wolf kill-rate per wolf, *P* is the wolf pack size, 30 is the number of days in a month, and *Q* is the percent of the edible biomass of a carcass consumed by a wolf pack given by Wilmers et al. [16]. We used Monte Carlo methods, as elaborated below, to reconstruct how much carrion would have been available to scavengers during each of the winter months (November through April) in the years 1950 and 2000 under scenarios with and without wolves. Specifically, for each scenario [1950 without wolves, 2000 without wolves, 1950 with wolves, and 2000 with wolves], we drew 100 random SDTH values for each of the months, where SDTH was assumed to be normally distributed with mean and standard error for the years 1950 and 2000 given by the regression analyses of the Tower Falls weather data (see Figure 2). This incorporated uncertainty into our estimate of SDTH for the years 1950 and 2000, allowing us to draw random SDTH values from those years for our Monte Carlo simulation. In the scenarios without wolves, we inserted our randomly chosen monthly SDTH values for each year and each run into equation 1 to yield the amount of carrion available per month without wolves. We used the same procedure for selecting SDTH in our scenario with wolves. In order to select wolf pack size, we assumed that wolf pack sizes were normally distributed, with a mean (± standard deviation) pack size of 10.6 (± 5) representing the current distribution of Yellowstone wolves [31]. We then inserted our randomly chosen monthly SDTH values and wolf pack sizes into equation 2 to yield the amount of carrion available per month with wolves. For each run of each scenario, we recorded the reduction in monthly winter biomass available to scavengers in 2000 as a proportion of what was available in 1950.

Our statistical modeling approach, although rooted empirically, is limited by the fact that it does not take into account the possible effects of wolf and elk population dynamics on carrion availability. In order to explore these effects, therefore, we used a previously published model [29] that was originally built to explore the effects of wolf and elk population dynamics on monthly carrion flow to scavengers.

#### Wolf effects: Dynamic model

The details of the model are exactly the same as in Wilmers and Getz [29], except for the following changes. In the original model, SDTH was incorporated into the elk population dynamics but was treated as a random variable. In the present study, we modified the model so that the actual progression of winter weather from 1950 to 2000 was used. We ran the model for 51 y, from 1950 to 2001. We selected SDTH, *V,* for the year and month in question from the Tower Falls regression equations in exactly the same manner that we describe above in the statistical model. Since the distribution of elk among age classes from 1950 is not known, we performed, as a baseline, a 50-y run of the model under average 1950 weather conditions. This is long enough for the effects of initial conditions to dissipate. We then used the numbers and age structure of the final month of the baseline run as the initial conditions of the run using observed weather data from 1950 to 2000.

Sensitivity analyses were conducted using Monte Carlo methods to assess the relative effects of different parameter values on model output [29,32]. Since the primary goal of using the dynamic model is to assess whether late-winter carrion will be affected by elk and wolf population dynamics in the context of a changing climate, we defined an output variable, *z,* as the percent change in late-winter carrion from 1950 to 2000. We assigned March and April to late winter for comparison to Figure 4, since these are the two months showing a significant effect between scenarios with and without wolves. For each scenario, we conducted 1,000 runs of the model, choosing a different set of parameter values at random from the ranges provided in Table 1 of Wilmers and Getz [29]. Each model parameter was then regressed against *z* to determine its effect.

## Abbreviations

NP: National Park
SDTH: snow depth
SNFL: snowfall
TMAX: maximum temperature
TMIN: minimum temperature
